# LRCH Proteins: A Novel Family of Cytoskeletal Regulators

**DOI:** 10.1371/journal.pone.0012257

**Published:** 2010-08-18

**Authors:** Hélène Foussard, Pierre Ferrer, Philippe Valenti, Cédric Polesello, Sébastien Carreno, François Payre

**Affiliations:** 1 Université de Toulouse UPS, Centre de Biologie du Développement, Toulouse, France; 2 CNRS, UMR5547, Centre de Biologie du Développement, Toulouse, France; The University of Hong Kong, Hong Kong

## Abstract

**Background:**

Comparative genomics has revealed an unexpected level of conservation for gene products across the evolution of animal species. However, the molecular function of only a few proteins has been investigated experimentally, and the role of many animal proteins still remains unknown. Here we report the characterization of a novel family of evolutionary conserved proteins, which display specific features of cytoskeletal scaffolding proteins, referred to as LRCHs.

**Principal Findings:**

Taking advantage of the existence of a single *LRCH* gene in flies, *dLRCH*, we explored its function in cultured cells, and show that dLRCH act to stabilize the cell cortex during cell division. *dLRCH* depletion leads to ectopic cortical blebs and alters positioning of the mitotic spindle. We further examined the consequences of *dLRCH* deletion throughout development and adult life. Although *dLRCH* is not essential for cell division *in vivo*, flies lacking *dLRCH* display a reduced fertility and fitness, particularly when raised at extreme temperatures.

**Conclusion/Significance:**

These results support the idea that some cytoskeletal regulators are important to buffer environmental variations and ensure the proper execution of basic cellular processes, such as the control of cell shape, under environmental variations.

## Introduction

The division of animal cells relies on the choreographed reorganization of the mitotic spindle, which is responsible for chromosome segregation. To ensure that the two daughter cells receive identical genomic complements, microtubule dynamics must be coordinated with a stereotyped series of changes in cell shape, leading to cytokinesis. A failure to coordinate cell shape transformations with chromosome separation can lead to aneuploidy and contribute to cancer [1]. At the onset of mitosis, reorganization of the actin cytoskeleton drives cell rounding and cortical stiffening in early prophase [2]. Most animal cells therefore display a characteristic round shape in metaphase, at the time when microtubules build the mitotic spindle. Subsequently, the extended spindle guides the assembly of an equatorial acto-myosin ring, which, by contraction, divides the cell into two at the end of telophase [3]. Compared to the numerous factors identified for their role in the assembly of the contractile ring [3], the mechanisms controlling the organization of the cortical cytoskeleton at earlier stages of mitosis remain poorly understood [2].

It is well established that ERM proteins, named after the vertebrate members Ezrin, Radixin and Moesin, link actin filaments to membrane proteins [4], [5] following an activation step that includes phosphorylation of a conserved Threonine residue [6]. Moesin (Moe) represents the unique *Drosophila* member of the ERM family and we and other have shown a role for Moe in regulating cortical stability and rigidity during mitosis [7], [8]. Indeed, Moe depletion in *Drosophila* cells destabilizes the cell cortex throughout mitosis, leading to cortical deformations and abnormal distribution of acto-myosin regulators [7], [8]. In addition, the lack of Moe impairs microtubule organization and precludes stable positioning of the mitotic spindle. Mitosis onset is characterized by a burst of Moe activation and the spatiotemporal regulation of Moe activity plays an important role in coupling cell shape control and spindle morphogenesis during mitosis [7], [8].

To further explore the mechanisms regulating cortical organization during mitosis, we searched for putative partners of Moe. A two-hybrid screen identified the product of a candidate gene, CG6860 (hereafter referred to as *dLRCH*), as a potential physical interactor of Moe [9]. We show here that dLRCH defines a novel family of proteins, contributing to cortical organization during cell division. dLRCH localizes at the cleavage furrow in ana/telophase, partly colocalizing with activated Moe. Depletion of *dLRCH* in *Drosophila* S2 cells causes short-lived blebs that deform the cortex during mitosis, as well as alteration of spindle positioning. However, flies lacking *dLRCH* develop to adulthood, showing that *dLRCH* activity is not essential for cell division *in vivo*. Nonetheless, *dLRCH* deficient flies are female sterile, display shortened longevity and reduced resistance to extreme conditions. Consistently with the evolutionary conservation of LRCH proteins in animals, this first functional analysis therefore supports that *dLRCH* is required for proper development and physiology of *Drosophila*.

## Results

### dLRCH defines a novel family of putative cytoskeletal regulators

**Figure 1:**
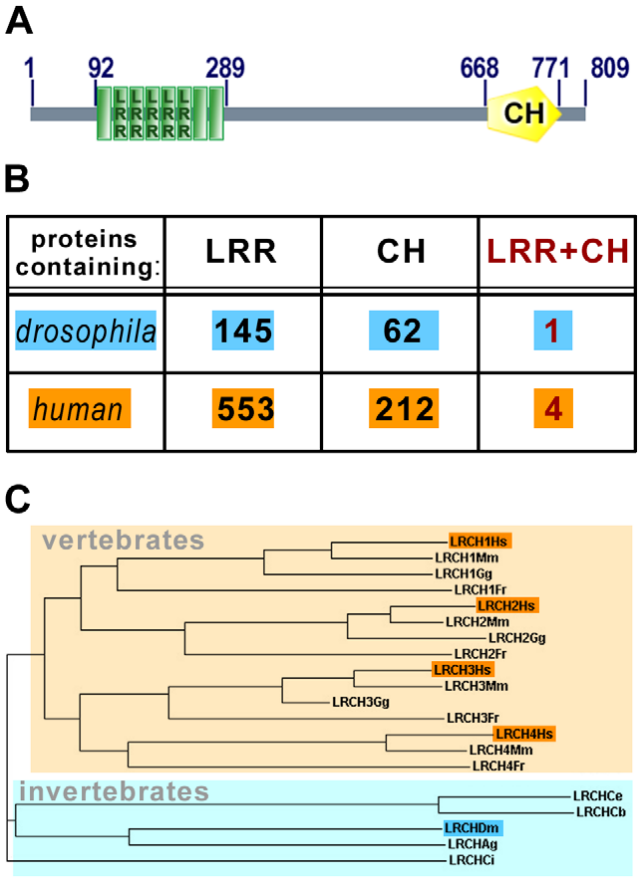
dLRCH defines a novel protein family evolutionary conserved in animals. **A**. The predicted *Drosophila* dLRCH protein includes two regions with recognizable motifs: eight repetitions of Leucine Rich Repeats (LRR) from position 92 to 289 and a Calponin Homology (CH) domain in the C-terminus (position 668 to 771). Domains that display a typical LRR organization are shown as LRR-labeled green boxes, degenerate LRR-like structure are shown as green boxes. **B**. Number of proteins that contain LRR, CH or LRR+CH domains in flies and humans. **C**. Distribution of LRCH proteins throughout different animal phyla. *Hs, Homo sapiens; Mm, Mus musculus; Gg, Gallus gallus; Fr, Fugu rubripes; Ce, Caenorhabditis elegans; Cb, Caenorhabditis briggsae; Dm, Drosophila melanogaster; Ag, Anopheles gambiae; Ci, Ciona intestinalis*.

CG6860 was identified in a genome-wide two-hybrid screen as being a potential Moe interactor [9]. Annotation of the *Drosophila* genome predicts that CG6860 encodes a novel protein of 809 amino acids (aa) that we named dLRCH, since it comprises Leucine-Rich-Repeats (LRR) and a Calponin Homology (CH) domain (Figure 1A). The N-terminal region harbors five LRR, a motif of 22–26aa defined by the consensus LxxLxLxxNxLxxLPxxL (where L can be leu, val, ile, or phe), previously shown to provide a structural framework for protein/protein interactions [10]. Careful examination revealed the existence of three additional motifs, partly matching the LRR consensus [11], which flank the five *bona fide* LRR. Furthermore, the C-terminal region of dLRCH is characterized by the presence of a CH domain, generally viewed as an actin-binding module [12].

Although LRR and CH domains are widespread in eukaryotes, the combination of both LRR and CH within a same protein appears restricted to animal species, and specific to dLRCH in flies (Figure 1B). In human, only four highly-related proteins (hLRCH1-4) simultaneously harbor these two motifs. hLRCHs display extensive sequence similarity with dLRCH (Figure S1), reinforcing the conclusion that they share a common evolutionary origin (Figure 1C). Taken together, these data show that dLRCH defines a novel family of proteins, whose patterns of conserved amino-acids suggest that they act as cytoskeletal scaffold factors.

### LRCH proteins localize at the cell cortex and cleavage furrow during mitosis

Recent work has highlighted the importance of the spatio-temporal localization and activity of cytoskeletal regulators during the successive steps of cell division [2], [7], [8]. As a first step in the analysis of LRCH proteins, we examined the sub-cellular distribution of dLRCH during mitosis.

**Figure 2:**
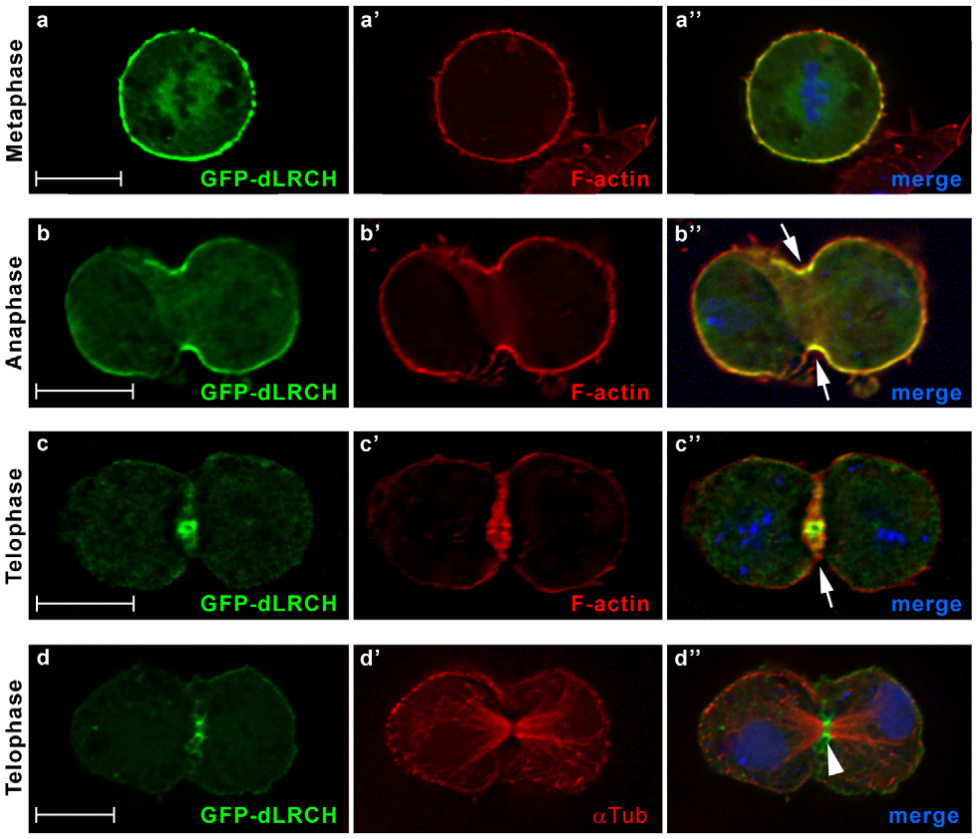
dLRCH accumulates at the mitotic cortex and cleavage furrow. Sub-cellular distribution of a GFP-dLRCH fusion protein throughout the successive stages of cell division, as assayed in cultured *Drosophila* S2 cells. (a–c″) Co-detection of GFP-dLRCH (green) and F-actin (red), from metaphase to telophase. In metaphase, dLRCH is enriched at the cell cortex, co-localizing with F-actin. Starting from anaphase, GFP-dLRCH accumulates at the cleavage furrow (arrows). During late telophase, GFP-dLRCH becomes concentrated at the midbody region (arrowhead) as shown in double labelings with α-tubulin (d–d″). DNA is in blue in merged images (a″, b″, c″ and d″).

**Figure 3:**
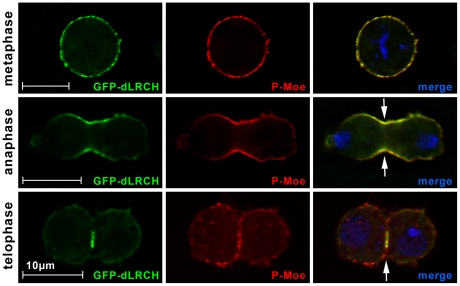
dLRCH parallels the distribution of activated Moe during cell division. The GFP-dLRCH fusion protein co-localizes with activated Moe during mitosis. Distribution of P-Moe (red) in dividing S2 cells shows a pattern reminiscent to GFP-dLRCH localization (green). Whereas in metaphase both proteins are detected around the entire cell cortex, GFP-dLRCH and P-Moe become restricted to the cleavage furrow (arrows) from anaphase to the end of mitosis. DNA is in blue.

*Drosophila* S2 cells were transfected with constructs encoding a GFP-tagged dLRCH protein, a tool that is also suitable for live imaging (see below). GFP-dLRCH is distributed at the cell cortex where it colocalized with the actin network, at the onset of mitosis (Figure 2a–a″). In addition, a weak signal was often detected associated with spindle microtubules in metaphase (Figure 2a and data not shown). In anaphase, cortical dLRCH became enriched at the equator (Figure 2b–b″) and then localized at the cleavage furrow until the end of mitosis (Figure 2c–d″). Antibodies specific for phosphorylated Moe (P-Moe) [13] showed that GFP-dLRCH overlaps with sites of Moe activation during cell division (Figure 3). dLRCH thus displays a dynamic sub-cellular distribution in mitotic cells, colocalizing with F-actin rich structures and activated Moesin.

### *dLRCH* depletion induces deformation of the mitotic cortex

The localization of dLRCH during cell division is suggestive of a role in the organization of the mitotic cortex. To examine this hypothesis, we knocked down *dLRCH* activity in S2 cells and analyzed its consequences on mitosis.

**Figure 4:**
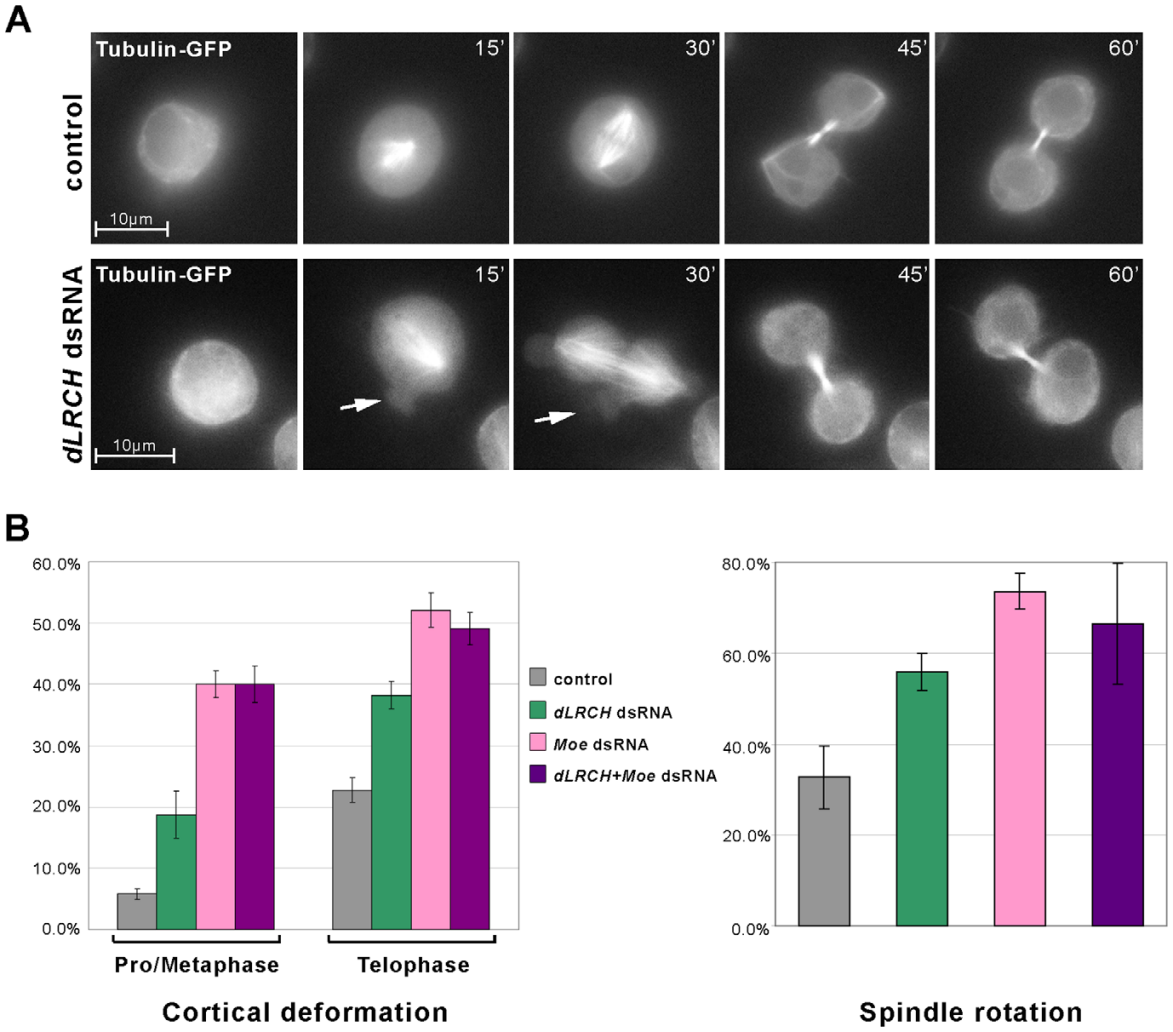
*dLRCH* depletion induces deformation of the mitotic cortex. **A**. Time-lapse frames of living S2 cells stably expressing Tubulin-GFP. Top panels shows a dividing S2 cell in control conditions (no dsRNA), lower panels shows a *dLRCH*-depleted cell displaying abnormal cortical protrusions (arrows) from pro/metaphase to ana/telophase. **B**. Quantification of the defects observed in cortical and spindle organization upon depletion of *dLRCH* and/or *Moe*. The left chart plots cortical deformation, with the presence of at least one bleb during cell division (with the exception of those normally observed at the polar cortex). Right panel shows the variation in spindle orientation after treatment with *dLRCH*, *Moe* or both dsRNA. Spindle rotation was estimated by measuring the angle of the axis of the spindle from the first metaphase observed to that of telophase (n = 329, 391, 280 and 182 cells for control, *Moe* dsRNA, *dLRCH* dsRNA and *Moe+dLRCH* dsRNA, respectively). Errors bars represent SD.

Results obtained from the use of two different dsRNA, targeting either the 5′ or 3′ region of the *dLRCH* ORF (Figure S2), indicate that the depletion of dLRCH impinges on cell shape during mitosis (Figure 4A). Live imaging using a stable line expressing Tubulin-GFP (Tub-GFP) showed that *dLRCH-*depleted cells displayed short-lived cytoplasmic bulges, or blebs, that transiently deformed the cortex (Figure 4A), visible from pro-metaphase stages. Cortical deformations were also seen in *dLRCH-*depleted cells at later stages, notably in the equatorial region that is normally not subjected to blebbing in control conditions (Figure 4A). Most *dLRCH*-depleted cells completed cell division and we observed only a limited increase of cytokinesis failure, as evaluated by the proportion of binucleated cells (1.1% +/-1.6 in controls and 4.8% +/−1.9 in *dLRCH*-depleted cells). We then compared the consequences of *dLRCH* and *Moe* inactivation during cell division. The depletion of either *dLRCH* or *Moe* led to significant cortical blebbing in pro/metaphase and ana/telophase, albeit with a lower proportion of cells displayed defects in *dLRCH-*depleted cells compared to those lacking Moe (Figure 4B). In addition, cortical blebs resulting from *dLRCH* depletion are rapidly retracted as seen in live-imaging (Figure 4A) and being hardly detected in fixed samples. The absence of *Moe* activity leads to longer lived deformations, easily seen in fixed samples [7], [8]. Interestingly, *dLRCH* depletion also led to improper positioning of the spindle (Figure 4B), as seen in living cells (Figure 4A). Again, the proportion of cells showing destabilized spindle was less pronounced following the depletion of *dLRCH* versus *Moe*. Therefore, during the division of S2 cells *dLRCH* depletion triggers *Moe*-like defects, albeit of weaker severity and frequency.

We therefore examined whether the distribution of each putative partner could depend on the function of the other. Upon *dLRCH* depletion, phosphorylated Moe was properly distributed at the cortex in metaphase (data not shown) and enriched at the cleavage furrow in anaphase cells (Figure S3A). Reciprocally, using S2 cells stably expressing GFP-dLRCH, we found that *Moe* or *Slik* activity is dispensable for the proper localization of dLRCH at the cleavage furrow (Figure S3B,C). These data therefore suggest that the distribution of dLRCH and Moesin relies on independent mechanisms during cell division. Consistent with this conclusion, we failed to detect the existence of a stable dLRCH/Moe complex in *Drosophila* cells or embryos, as assayed by co-immuno-precipitation (data not shown). The various defects observed during the division of *dLRCH*-depleted cells, *i.e.* cortical blebs and spindle mis-positioning, are however likely to involve Moe, since the simultaneous depletion of *dLRCH* and *Moe* did not lead to any aggravation of the defects exhibited in the absence of Moe (Figure 4B).

### Flies carrying a deletion of the *dLRCH* locus display female-specific sterility

Taken together, our results obtained in cultured cells showed that dLRCH contributes to the control of cell division. We next investigated the function of *dLRCH in vivo*, taking advantage of the existence of a single representative gene in *Drosophila*.

**Figure 5:**
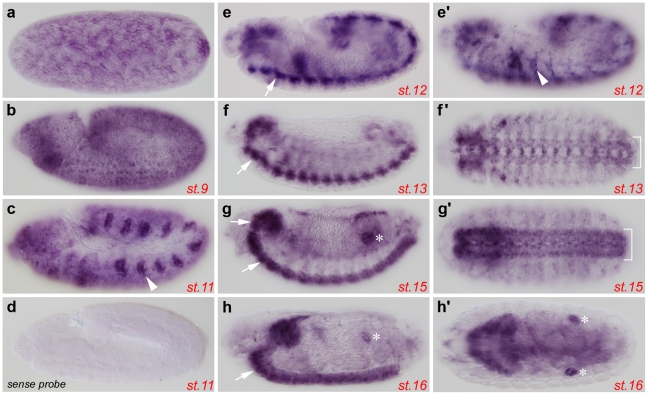
Expression pattern of *dLRCH* during *Drosophila* embryogenesis. Embryonic expression of *dLRCH* is ubiquitous and reinforced in specific tissues, as shown by whole mount *in situ* hybridization to *dLRCH* mRNA. (a–h, e′) Pictures correspond to lateral views of embryos from early to late embryonic stages or (f′–h′) to dorsal views of embryos from stage 13 to 16. Besides a low-level ubiquitous expression, higher levels of *dLRCH* mRNA accumulates in tracheal pits (arrowheads in panels c and e′), the central nervous system (arrows in panels e, f, g, h, brackets in f′ and g′), as well as in embryonic gonads (stars in panels g, h and h′). (d) The picture shows the staining obtained with a control sense probe.

We first examined the expression pattern of *dLRCH* throughout embryonic development using *in situ* hybridization (Figure 5). *dLRCH* RNA was detected from early stages of embryogenesis, suggestive of a maternal contribution to *dLRCH* embryonic expression. Following the onset of zygotic transcription, *dLRCH* RNA was expressed ubiquitously. Nevertheless, *dLRCH* expression was reinforced in several embryonic tissues. From stage 11 to 12, *dLRCH* RNA accumulated in the developing tracheal system (Figure 5c,e′), then in subsets of cells composing the Central Nervous System (Figure 5e–g′). Finally, from stage-15, *dLRCH* was also strongly expressed in the gonads (Figure 5g–h′).

**Figure 6:**
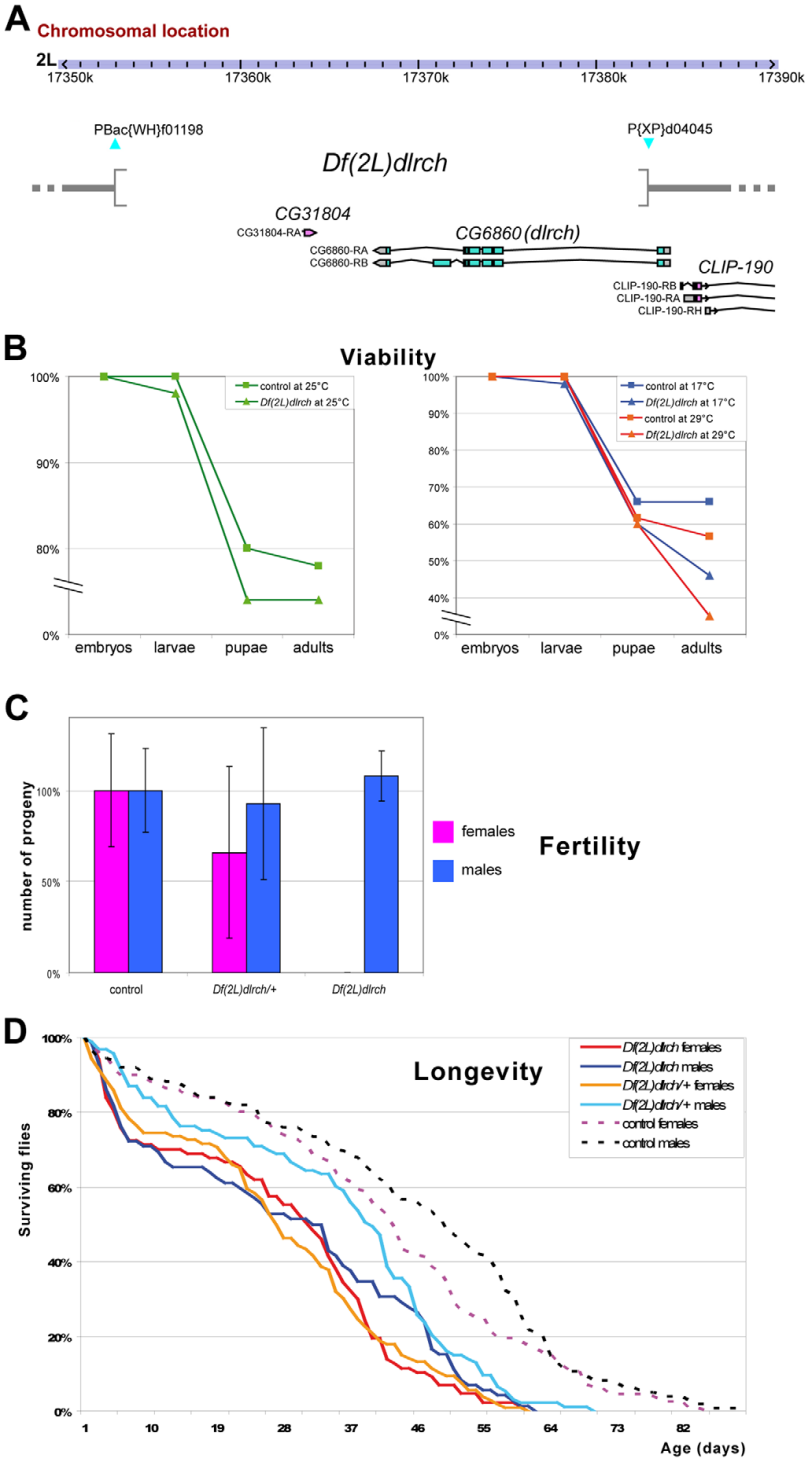
Consequences of the lack of *dLRCH* on development and lifespan. **A**. Genomic organization of the *dLRCH* (*CG6860*) locus, with coding exons drawn in light blue. The *Df(2L)dLRCH* was generated by provoking recombination between two transposons carrying FRT sites (Pbac(WH)f01198 and P(XP)CG6860[d04045]) and its molecular structure was verified by PCR. *Df(2L)dLRCH* removes most *dLRCH* coding sequence, but the first exon which encodes 101 aa. The 5′ breakpoint of *Df(2L)dLRCH* is located 1.8 kb upstream of CLIP-190 and thus not likely to interfere with CLIP-190 expression, although the latter point remains to be tested experimentally. **B**. Survival rate of *Df(2L)dLRCH* homozygous individuals throughout development, at 25°C (left panel, n = 50 embryos), 17°C or 29°C (right panel, n = 50 and 60, respectively). The parental line P[XP]d04045 was used as control. **C**. The fertility was evaluated as the number of adult progeny emerging from crosses between a control *w* strain and *Df(2L)dLRCH* mutants (n = 872, 575, 0 for control females, heterozygous and homozygous *Df(2L)dLRCH*; and n = 872, 808 and 752 for males of the corresponding genotypes). All values were compared to control crosses (100%). Errors bars represent SD. **D**. Longevity of adult flies submitted to 25°C conditions. n = 160 for control females, n = 161 for control males, n = 106 for *Df(2L)dLRCH* heterozygous females, n = 93 for *Df(2L)dLRCH* heterozygous males, n = 87 for *Df(2L)dLRCH* homozygous females and n = 72 for *Df(2L)dLRCH* homozygous males.

We generated a loss of function allele by inducing a short genomic deletion, using FRT-mediated recombination between two transposable elements [14] that flank the *dLRCH* locus (Figure 6A). This deletion, called *Df(2L)dLRCH*, removes ∼30 kb that include most of *dLRCH* coding regions, as well as *CG31804*, a predicted gene showing very poor evolutionary conservation (even within *Drosophila* species, see Figure S4) and no evidence of expression in embryos and females [15]. That *Df(2L)dLRCH* represents a null *dLRCH* allele was confirmed by genomic PCR, with homozygous mutant embryos (Figure S2B). We found that embryos homozygous for *Df(2L)dLRCH* were viable and did not display gross developmental defects. Accordingly, it has been reported that a transposable element inserted in *dLRCH* exonic sequences, CG6860^T1–36C^, does not affect viability [16]. *Df(2L)dLRCH* mutants developed to adult stage, albeit with a slightly decreased viability when compared to wild-type (Figure 6B). While adult males lacking *dLRCH* are fully fertile, we observed a strong reduction in female fertility. Firstly, females heterozygous for *Df(2L)dLRCH* displayed decreased fertility when compared to their sibling controls (Figure 6C). Moreover, *Df(2L)dLRCH* homozygous females were sterile (Figure 6C), laying rare embryos that displayed no sign of development, as deduced from the absence of detectable nuclear division. While most egg chambers from *Df(2L)dLRCH* females appeared normal, we observed a weakly penetrant phenotype characterized by an abnormal number of germ cells (Figure S5). Although this observation cannot explain the 100% penetrant phenotype of female sterility, these defects might suggest a role for *dLRCH* in the control of germ cell division.

The slightly reduced viability of *Df(2L)dLRCH* mutants opened the possibility that the absence of *dLRCH* impinges on development and physiology. We reasoned that if the activity of *dLRCH* is not essential under optimal culture conditions, its absence might be further deleterious under conditions of stress. We examined whether either low or high temperature, *i.e.*, 17°C or 29°C that are close to the extreme allowing *Drosophila* development, impact on the survival of *Df(2L)dLRCH* mutants when compared to control. At these temperatures, we observed that *Df(2L)dLRCH* mutants displayed increased lethality (Figure 6B), indicating that *dLRCH* activity contributes to the robustness of the fly when placed in harsh environmental conditions. This conclusion was further supported when examining the longevity of individuals. Even when raised in optimal conditions, *Df(2L)dLRCH* mutants exhibited a reduced longevity in both sexes, already detectable in heterozygous conditions (Figure 6D). Interestingly, *Df(2L)dLRCH* females showed the most marked effect, even though reducing female fecundity is often thought to have a positive impact on lifespan [17]. Taken together, these *in vivo* results show that whereas *dLRCH* function is not essential in flies, its absence significantly affects the fitness of animals, especially under non-optimal conditions of development and during adult life.

## Discussion

We identified here a novel family of putative cytoskeletal scaffold proteins, referred to as LRCH. Our studies in *Drosophila* cells implicate LRCH proteins in the organization of the cortex during cell division. dLRCH protein localizes to cortical regions undergoing contraction and depletion of *dLRCH* consistently provokes defects in both cell shape and positioning of the spindle during mitosis. However, the defects observed in cultured cells do not prevent a coherent development, since embryos lacking *dLRCH* reach adulthood. Nevertheless, our genetic analyses indicate that *dLRCH* activity is likely to be required for optimal resistance of individuals facing environmental constraints and aging.

### LRCH proteins define a novel family of cytoskeletal factors

LRCH proteins are characterized by a unique combination of protein domains that are otherwise common in eukaryotes, the LRR and CH domain. The distinctive domain organization of dLRCH defines a novel family of proteins, strongly conserved throughout the evolution of animals. Indeed, the human genome, as well as that of other mammals, contains four highly related *LRCH* genes. Calponin Homology domains (often arranged in 2–4 tandem repetitions) are well established as protein domains mediating interaction with actin filaments [12]. Further studies have shown that five different subtypes of CH domain can be discriminated, some of which are involved in binding to microtubules [18], [19]. The CH domain of LRCH proteins belongs to the type-3 class [18], suggesting that, like the CH of IQGAP1 [20], it mediates interaction with the actin network; a conclusion consistent with LRCH colocalization with actin-rich regions of the cortex during mitosis. LRR motifs display a rapid diversification outside their structural backbone and have been implicated in protein/protein interactions [10], [11], [21], but their structure does not allow the prediction of physical partners of LRCH proteins. While large-scale two hybrid screenings in yeast have suggested that LRCH might bind to ERM proteins [9], we did not detect such an interaction in cultured cells or within the whole organism. In addition, our results show that the subcellular localization of Moe and dLRCH are mutually independent in dividing S2 cells.

While the LRCH family of proteins is well conserved in animals, little is known concerning their role in development or physiology. A few genetic studies have suggested that a variant in hLRCH1 is associated with knee osteoarthritis [22], [23], [24] and recently an hLRCH3 polymorphism was found to be associated with susceptibility alleles to E. coli F4ab/F4ac in pigs [25]. Besides these sparse functional indications, nothing is known about the molecular activity of LRCH proteins.

### LRCH proteins function at the cortex of mitotic cells

The sub-cellular localization of dLRCH during mitosis supports the idea that LRCH proteins function as cytoskeletal regulators during cell division. dLRCH localizes at the actin-rich cell cortex, where it partly overlaps with P-Moe throughout cell division [7], [8]. We used live cell imaging to follow cortical organization in *dLRCH*-depleted cells as they passed through mitosis. *dLRCH*-depleted cells display abnormal transient cortical protrusions, or blebs, indicating that dLRCH contributes to cell shape control during mitosis. The formation of blebs results from a localized rupture of the interaction between actin filaments and the plasma membrane [26], [27], leading first to membrane expansion, then rapidly followed by blebs retraction upon reassembly of the actin cortex. Blebs occur in a number of instances during normal cell physiology such as cytokinesis [28], where they are formed specifically at the poles during anaphase [29], thereby contributing to the so-called polar relaxation that facilitates cell elongation. In contrast, the abnormal blebs of *dLRCH*-depleted cells form from early stages of mitosis and occur notably in the equatorial region at later stages. It has been shown that ERM proteins are normally recruited during the earliest steps of bleb retraction in normal cells, and Ezrin is required to retract short-lived blebs induced by the alteration of cortical tension in mammalian cells [26], [30], [31]. Since *dLRCH*-depleted cells show weak *Moe*-like defects during mitosis, LRCH proteins might contribute to proper organization of the mitotic cortex. We propose that the lack of LRCH activity causes transient alterations of the cell cortex, which are rapidly corrected in the presence of Moe, explaining why these abnormal blebs are only short-lived, as well as the absence of additive defects following the simultaneous depletion of both dLRCH and Moe.

Several lines of evidences suggest that, like in flies, LRCH and ERM proteins are also involved in the control of cell division in mammals. First, ERM and LRCH proteins show similar localization during the division of HeLa cells (see Figure S6). Human ERM proteins are strongly activated upon mitosis onset, accumulating at the cortex in pro/metaphase, then becoming restricted to the cleavage furrow. The three ERM proteins accumulate at the furrow, thus each can participate in the control of cell division (Figure S6B). Similarly, we found that hLRCH3 accumulates at the cleavage furrow from anaphase onset (Figure S6C), albeit without significant cortical accumulation being observed at previous stages of mitosis. Secondly, genome-scale RNAi profiling has shown that depletion of hLRCH2 and hMoe alter the division of HeLa cells [32]. Since the three ERM proteins (and presumably the four hLRCH proteins) likely play, at least partly, redundant roles in the control of cell division, the individual inactivation of hLRCH2 and hMoe probably underestimates the consequences of the lack of ERM activity on the one hand, or LRCH activity on the other hand. Finally, recent studies in human cells shows that hLRCHs belong to the subset of proteins being specifically phosphorylated during mitosis [33], in a cell cycle dependent manner. This provides further evidence that LRCH activity, and its putative regulation by phosphorylation, may play a role in the control of cell division.

### Towards a role of LRCHs in the mitotic spindle organization

Live imaging revealed the instability of spindle orientation in the absence of dLRCH during mitosis. Such a defect likely reflects an alteration of the cross-talk that normally occurs between the cortical actin network and microtubule tips during cell division [3], [34] and that requires Moe function [7], [8].

It remains yet possible that LRCH proteins contribute more directly to microtubule organization during cell division. It is worth noting that EB1, a protein binding microtubule plus-ends through a different subtype of CH domain, is required for proper positioning of the mitotic spindle [19], [35]. While the individual CH domain of LRCH proteins is unlikely to interact with microtubules, it has been shown that two CH domains brought together by homo- (*e.g.*, EB1) or hetero-dimerisation (*e.g.*, Ncd80 and Nuf2) form a microtubule binding unit [19], [36]. Interestingly, recent studies have shown that ERM proteins impact on the organization of specific subsets of microtubules in various cell types [37], [38], [39]. Therefore, both ERM and LRCH proteins might have the potential to influence the coordination between cortex and mitotic spindle through both F-actin and microtubule organization.

### *In vivo* analysis of LRCH activity

Functional assays in cultured cells have been proven as an efficient means of identifying the molecular pathways involved in the control of both cell shape [40] and division [41], [42]. Nevertheless, to which extent cell-based assays can predict the functional implication of a given factor within the whole organism remains a debated matter. To assess the function of *dLRCH in vivo*, we generated a loss of function *dLRCH* allele in flies, by inducing a targeted genomic deficiency that removes only *dLRCH* and a neighboring predicted gene (*CG31804*); *CG31804* is poorly evolutionary conserved, even in closely related species, and both micro-array and deep sequencing experiments have not revealed expression, aside weak levels in the adult testis (Flybase website (accessed 2010) http://flybase.org/). If an influence of CG31804 cannot be formally ruled out, these results collectively suggest that the phenotype observed in *Df(2L)dLRCH* is primary due to the absence of *dLRCH* activity. In any case, embryos lacking *dLRCH* progress through an apparently normal development, showing that *dLRCH* is dispensable for cell division *in vivo*. Furthermore, it is well known that the division of spermatids in males is particularly sensitive to alterations in the molecular mechanisms controlling mitosis [43], [44]. Indeed, several mutations that do not prevent the division of somatic cells result in male sterility, due to improper spermatid division. The full fertility of *Df(2L)dLRCH* males further argues that cytokinesis occurs normally in the absence of *dLRCH in vivo*.

Similarly, it was recently shown that despite the dramatic defects observed in dividing S2 cells, the absence of Wac, a component of the Augmin complex, does not affect *Drosophila* development and leads to fully fertile males, but sterile females [45]. In this case, while Wac is not essential to mitosis *in vivo*, its function is indeed required for chromosome alignment and segregation during female meiosis. Interestingly, adult females lacking *dLRCH* are sterile and even *Df(2L)dLRCH* heterozygous display a phenotype of hypo-fertility. These data therefore open the possibility that *dLRCH* might also be involved in female meiosis, a speculation that must await future experimental investigation for confirmation.

Phenotypic characterization is a difficult task that often limits the functional outcomes of reverse genetic approaches. If evolutionary conservation clearly represents a signature of molecules that might be involved in important developmental mechanisms, the selective pressure applied both on efficient reproduction and adult fitness, two parameters rarely examined in cellular and developmental studies. For example, it has been shown that the conserved miRNA miR7 is not essential for the development of *Drosophila* raised under optimal culture conditions, but is required to maintain sensory organ fate under fluctuating temperature conditions [46], providing an important aspect of phenotypical robustness for developmental programs. In a same vein, we find that the absence of *dLRCH* negatively impacts on resistance of flies to extreme temperature conditions, as well as longevity. In addition to apparently redundant transcriptional enhancers [47], these data suggest that conserved cytoskeletal scaffolding proteins may fulfill buffering functions. Since temperature influences membrane fluidity, enzymatic activity and protein-protein interactions, scaffolding proteins might be important to stabilize the molecular networks that act at the cortex to control cell shape.

## Materials and Methods

### Sequence analysis

Putative structure of the dLRCH protein (FBpp0080521) was predicted by analyzing its sequence with the SMART package (http://smart.embl.de/). To determine the evolutionary repartition of proteins containing LRR and/or CH domains, Ensembl database (*Homo sapiens*) and FlyBase (*Drosophila*) were screened via Ensembl or Smart for PFAM domains (LRR: PF00560, and CH: PF00307). Individual sequences were extracted from Ensembl database: *Homo sapiens* (LRCH1 ENSP00000374448, LRCH2 ENSP00000360996, LRCH3 ENSP00000399751, LRCH4 ENSP00000309689), *Mus musculus* (LRCH1 ENSMUSP00000086361, LRCH2 ENSMUSP00000033647, LRCH3 ENSMUSP00000023491, LRCH4 ENSMUSP00000031734), *Fugu rubripes* (LRCH1 ENSTRUP00000007434, LRCH2 ENSTRUP00000042888, LRCH3 ENSTRUP00000006183, LRCH4 ENSTRUP00000004080), and *Ciona intestinalis* (ENSCINP00000002827), NCBI: *Gallus gallus* (LRCH1 XM_417050.2, LRCH2 XM_420210.2, LRCH3 XM_422732.2), WormBase: *Caenorhabditis elegans* (C14F11.2), and *Caenorhabditis briggsae* (CBP28441), FlyBase: Drosophila melanogaster (FBpp0080521) and Vector Base: *Anopheles gambiae* (AGAP010012-PA). Amino-acid sequences were aligned using CLUSTALW2 and evolutionary relationships viewed on a phylogram tree.

### Cell culture, dsRNA treatments and molecular constructs

*Drosophila* S2 cells were cultured in Schneider's medium (Invitrogen) supplemented with 10% heat-inactivated Fetal Bovine Serum (FBS) and Penicillin-Streptomycin (PS). dsRNA were synthesized and purified according to the T7 RiboMAX™ Express Large Scale RNA Production System (Promega). For *dLRCH* extinction, we used two non-overlapping regions to avoid off-target effects. For *Moe* and *Slik* extinction, we used a previously reported dsRNA [7]. dsRNA were added directly to cells growing in supplemented medium at the 1^st^, 3^rd^ and 6^th^ day of culture. Immuno-fluorescence assays or live imaging were performed at the 6^th^ day.

*dLRCH* sequences were subcloned into pEFGP-C1 that contains an actin promoter. Coding sequence of Ezrin, Radixin, Moesin on one hand, and hLRCH3 on the other hand were cloned into pEGFP-N1 or pEGFP-C1, respectively. Cells were transfected with GFP-dLRCH or GFP-hLRCH3-expressing plasmids using Fugen HD and immuno-fluorescence analyses were performed 60 hours after transfection. A stable cell line expressing GFP-dLRCH was established by co-transfection of GFP-dLRCH and Hygromycin resistance plasmids.

For immuno-fluorescence analysis, cells were cultured for 6 hours on glass coverslips, fixed in 4% paraformaldehyde in Schneider's supplemented medium (except for P-Moe and P-ERM, in which the cells were fixed in 10% TCA in FBS-free Schneider's medium), during 30 min. Cells were blocked with 2% BSA and 0.02% Saponin in TBS during one hour and then incubated with primary antibodies. We used anti-αTubulin (Sigma-Aldrich) at 1∶200, anti-γTubulin [48] at 1∶200, anti-Phospho-Moe [13] at 1∶100, and anti-Phospho-Ezrin (Thr567)/Radixin (Thr564)/Moesin (Thr558) Antibody (Cell Signalling) at 1∶100. Texas Red-X phalloidin (Invitrogen) was used at 1∶200 for F-actin staining. AlexaFluor488 secondary antibodies (Invitrogen) were used at 1/500. Texas Red dye-conjugated secondary antibodies (Jackson ImmunoResearch) were used at 1∶100.

### Imaging of fixed samples and time-lapse recording

Fixed cells were mounted in Vectashield containing DAPI (Vector Laboratories). Images were acquired using a Nikon Eclipse 90i microscope with a MicroMAX (Princeton Instruments) camera. Deconvolution was performed using Huygens software (Scientific Volume Imaging). Live cell imaging was performed using stable cell lines expressing Tubulin-GFP [7], or GFP-dLRCH, cultured in 96-well glass-bottom plates (Greiner) at 25°C and imaged with an inverted microscope (DMIRE2; Leica) equipped with a CoolSNAP HQ2 (Roper Scientific) camera and controlled by the MetaMorph 6.2 software (MDS Analytical Technologies). Spindle rotation in live cells was analyzed using ImageJ software. Images shown are representative of phenotypes observed in at least three independent experiments. All images were prepared using Photoshop (Adobe).

### In situ hybridization and fly strains

Dig-labeled sense and antisense probes were synthesized from a full length CG6860 cDNA, and used for *in situ* hybridization experiments following standard procedures. Embryos were mounted in glycerol-containing medium and photographed with a Nikon Eclipse 90i microscope. A null allele for *dLRCH* was generated through a small and targeted deletion using FLP recombinase [14] between the elements carrying FRT sites *f01198* and *d04045* obtained from Exelixis [49]. We kept four *Df(2L)dLRCH* stocks coming from independent chromosomal events for further analyses (Figure S2). The resulting deficiencies were characterized molecularly by PCR (Figure S2) using transposon-specific primers according to [14]. *Df(2L)dLRCH* mutant flies were kept and selected using GFP-expressing CyO balancers. The *w^1118^* strain, or the parental *d04045* line, was used as control, and the *y,w,Moe^PL106^/FM0* [50] line as *Moe* mutant.

### Viability and longevity experiments

For experiments aiming at estimating viability, eggs were collected from well-fed females kept on agar medium with drops of live yeast. After hatching, 50–60 larvae were transferred in polystyrene vials containing corn/yeast agar medium and allowed to develop in different incubators (at 17°C, 25°C or 29°C), where light was provided from 8am to 8pm. The longevity of adult flies was recorded using new born flies passed into fresh vials containing the same medium and a drop of live yeast, in an incubator at 25°C with an identical light/dark regime. The number of dead flies was recorded each day, up to death of the last fly. Vials were renewed twice a week.

## Supporting Information

Figure S1 Evolutionary conservation of Drosophila and human LRCH proteins. Alignments of protein sequences corresponding to the LRR motifs (A) and the CH domain (B) between dLRCH and its 4 human orthologs. The two respective regions (positions 79–281 and 669–772 within dLRCH) were aligned using ClustalW. Identical residues are in red; those in green and blue are highly or weakly similar across the five sequences, respectively. C. Schematic representation of conservation levels between Drosophila and human LRCH proteins. Each bar (arrow) represents a 10 aa window, displaying from 5–10 (black) or 3–4 (grey) identical residues in the five LRCH proteins. White bars indicate a poor conservation, with 0–2 invariant residues. Positions of the two functional domains are underlined.
(8.00 MB TIF)

Figure S2 Inactivation of dLRCH in cultured cells and whole animals. A. Living S2 cells stably expressing GFP-dLRCH in control conditions (no dsRNA or control dsRNA targeting Sip1) or treated with dsRNA targeting the N-term or C-term region of dLRCH ORF (dLRCH-N or dLRCH-C, respectively). Pictures were taken using the same exposure conditions. GFP signal is strongly reduced in cells treated with dLRCH-N & -C dsRNA but not in cells treated with control dsRNA. B. Genomic DNA extracted from single homozygous flies corresponding to independent recombination events was used as a template for PCR amplification, using primer specific for Sip1 (control) or dLRCH coding regions. No dLRCH amplification was observed in flies homozygous for the Df(2L)dLRCH, confirming that this represents a molecular null allele.
(2.88 MB TIF)

Figure S3 Reciprocal independence of dLRCH and the Moe pathway. A. Depletion of dLRCH does not affect P-Moe (red) distribution in dividing S2 cells, as shown by a normal accumulation of P-Moe at the equatorial cortex (arrows) during anaphase (in 91.4% of control cells, n = 58, and in 90% of dLRCH-depleted cells, n = 60). DNA is in blue B. Time-lapse frames of GFP-dLRCH S2 cells in control (left), and after treatment with Moe dsRNA (right) show that, reciprocally, Moe depletion does not prevent the proper distribution of dLRCH, as shown by accumulation at the cleavage furrow (arrow) in 98.0% of control cells and 97.7% of Moe-depleted cells, n = 300. C. Time-lapse frames of GFP-dLRCH S2 cells show that Slik depletion does not prevent dLRCH localization at the cleavage furrow (arrow), with GFP-dLRCH accumulating at furrow in 93.3% of control cells and 92.0% of Slik-depleted cells, n = 150. D. The graph shows the proportion of the different genotypes, observed in the progeny from MoePL106/FMO females crossed with control or Df(2L)dLRCH homozygous males. The absence of a dLRCH allele does not modify the proportion of the different classes, including that of MoePL106 male escapers, when compared to control. The number of individuals counted was MoePL106/+: n = 641; MoePL106/+; Df(2L)dLRCH/+: n = 680; FM0/+: n = 543; FM0/+; D(2L)dLRCH/+: n = 541; MoePL106/Y: n = 12; MoePL106/Y; Df(2L)dLRCH/+: n = 8; FM0/Y: n = 397; FM0/Y; D(2L)dLRCH/+: n = 421.
(5.57 MB TIF)

Figure S4 Evolution of the putative CG31804 gene in insect species. CG31804 putatively encodes a 212 amino-acid long peptide, being evolutionary conserved only in species of the melanogaster subgroup (grey box). Identities with the CG31804 encoded peptide decrease from 77% (D. simulans) to 57% (D. erecta). In the D. Ananassae genome, a genomic region at approximately the same location was identified by DNA similarity, but the presumptive ORF is interrupted by several stop codons and peptides do not display homology with the CG31804 product deduced from species of the melanogaster subgroup. Corresponding genome regions in other related drosophila were analysed with alignment tools (Blast, ClustalW2), which detect no significant DNA nor protein similarities.
(6.46 MB TIF)

Figure S5 Germline defects observed in Df(2L)dLRCH females. A. Quantification of the number of nurse cells in the egg chambers of control or Df(2L)dLRCH homozygous individuals, in 7 independent experiments. Control (w1118) flies showed a weak proportion of abnormal numbers of nurse cells (<15, or >15, per egg chamber), in all experiments. In contrast we observed high variability in the proportion of abnormal egg chambers in Df(2L)dLRCH homozygous females. In two separate experiments, these defects were seen in 60% of examined samples. B. Pictures of egg chambers dissected from control (left) and Df(2L)dLRCH (right) females illustrating the observed defects in the number of nurse cells. Nuclei (blue) were stained by Dapi and F-actin (red) by Phalloidin-Texas-Red.
(3.36 MB TIF)

Figure S6 Expression and localization of human ERMs and LRCH3 in Hela cells. A. RT-PCR experiments for each of the 3 ERM and the 4 hLRCH mRNA show that all seven genes are expressed in HeLa cells. B. Sub-cellular localization of Ezrin-GFP, Radixin-GFP or Moe-GFP (green) and F-actin (red) during telophase indicates that all three human ERM proteins are located at the cleavage furrow (arrows). C. At the end of mitosis, GFP-hLRCH3 (green) is detected at the cleavage furrow as shown in co-labeling with α-Tubulin or P-ERM (red). DNA is in blue in merged images.
(2.02 MB TIF)
