# Supplementary figures and images for: LRCH Proteins: A Novel Family of Cytoskeletal Regulators
Source: PLoS One. 2010 Aug 18;5(8):e12257. doi: 10.1371/journal.pone.0012257 (PMC2923620; doi:10.1371/journal.pone.0012257)

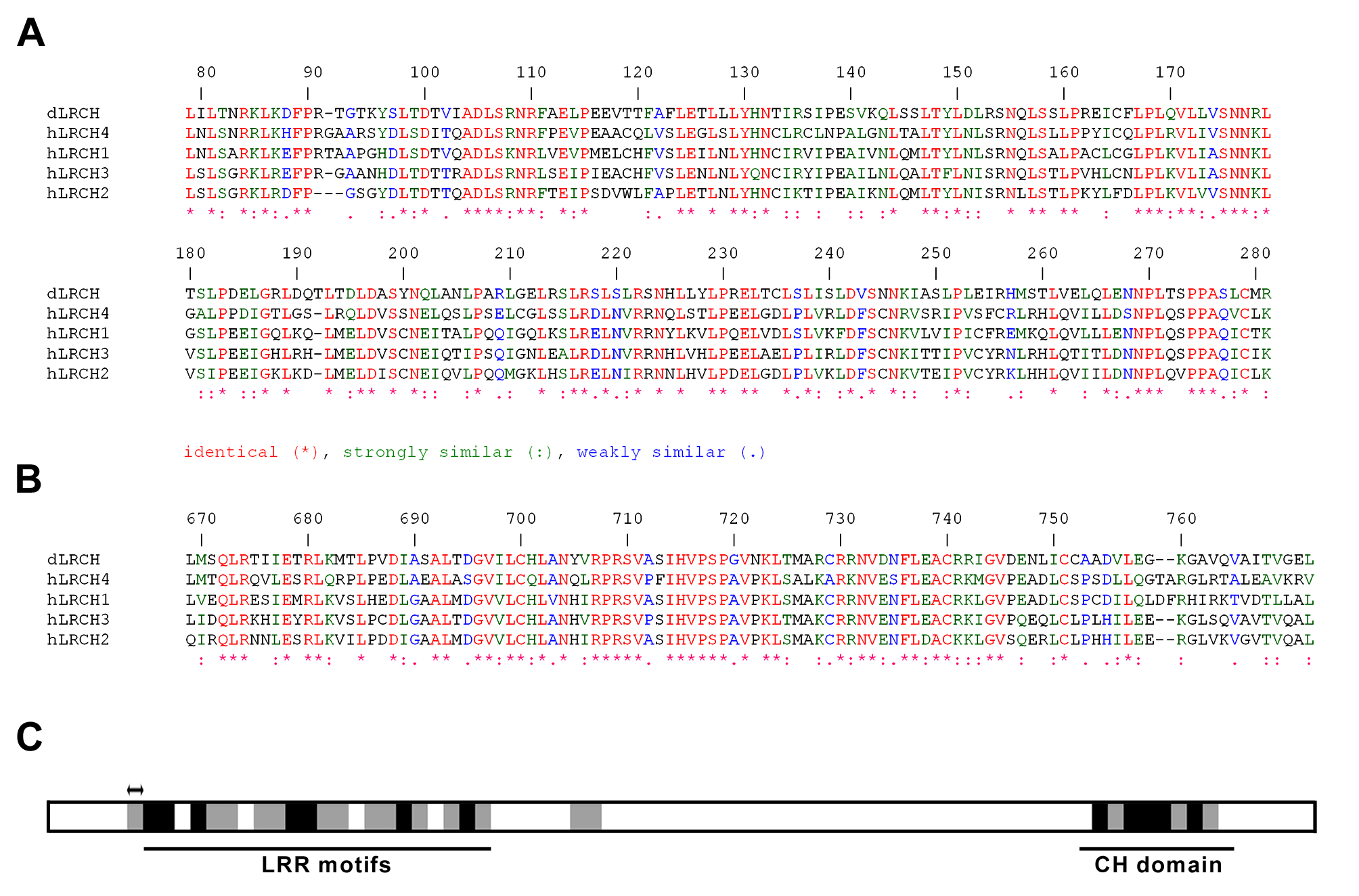

Supplement: Figure S1 — Evolutionary conservation of Drosophila and human LRCH proteins. Alignments of protein sequences corresponding to the LRR motifs (A) and the CH domain (B) between dLRCH and its 4 human orthologs. The two respective regions (positions 79–281 and 669–772 within dLRCH) were aligned using ClustalW. Identical residues are in red; those in green and blue are highly or weakly similar across the five sequences, respectively. C. Schematic representation of conservation levels between Drosophila and human LRCH proteins. Each bar (arrow) represents a 10 aa window, displaying from 5–10 (black) or 3–4 (grey) identical residues in the five LRCH proteins. White bars indicate a poor conservation, with 0–2 invariant residues. Positions of the two functional domains are underlined. (8.00 MB TIF) [file pone.0012257.s001.tif]

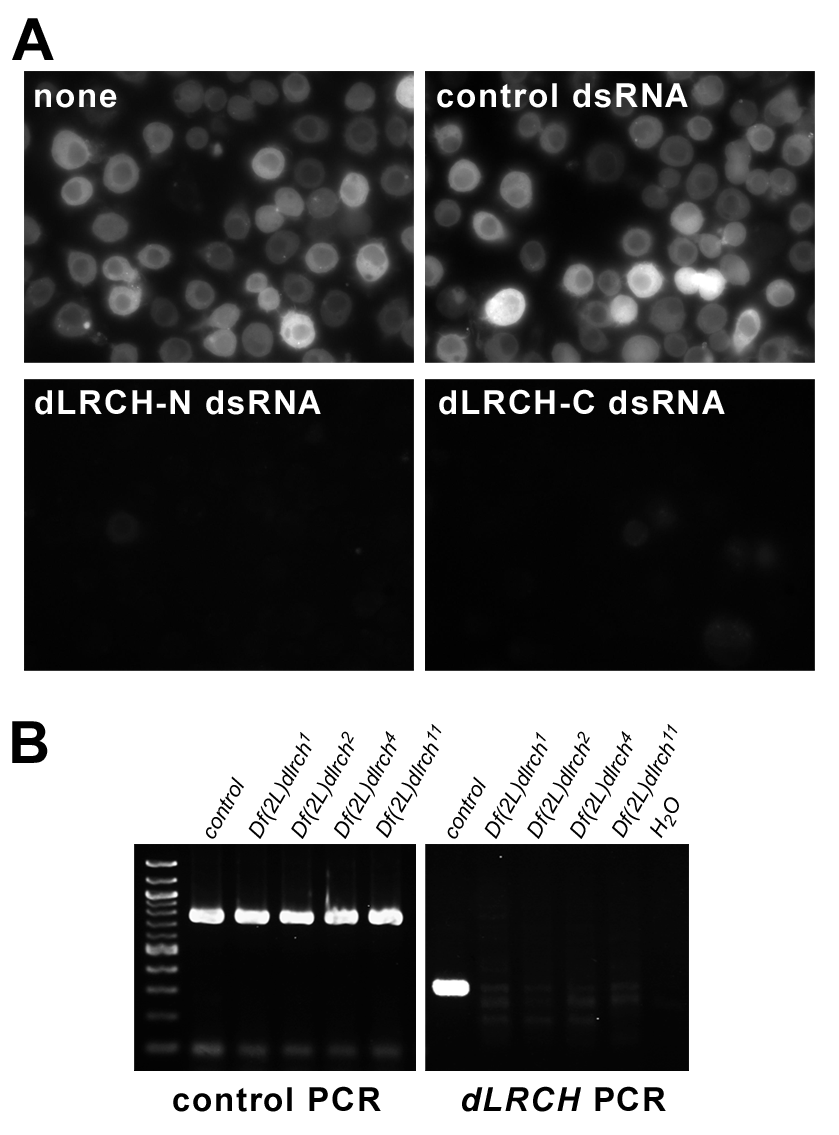

Supplement: Figure S2 — Inactivation of dLRCH in cultured cells and whole animals. A. Living S2 cells stably expressing GFP-dLRCH in control conditions (no dsRNA or control dsRNA targeting Sip1) or treated with dsRNA targeting the N-term or C-term region of dLRCH ORF (dLRCH-N or dLRCH-C, respectively). Pictures were taken using the same exposure conditions. GFP signal is strongly reduced in cells treated with dLRCH-N & -C dsRNA but not in cells treated with control dsRNA. B. Genomic DNA extracted from single homozygous flies corresponding to independent recombination events was used as a template for PCR amplification, using primer specific for Sip1 (control) or dLRCH coding regions. No dLRCH amplification was observed in flies homozygous for the Df(2L)dLRCH, confirming that this represents a molecular null allele. (2.88 MB TIF) [file pone.0012257.s002.tif]

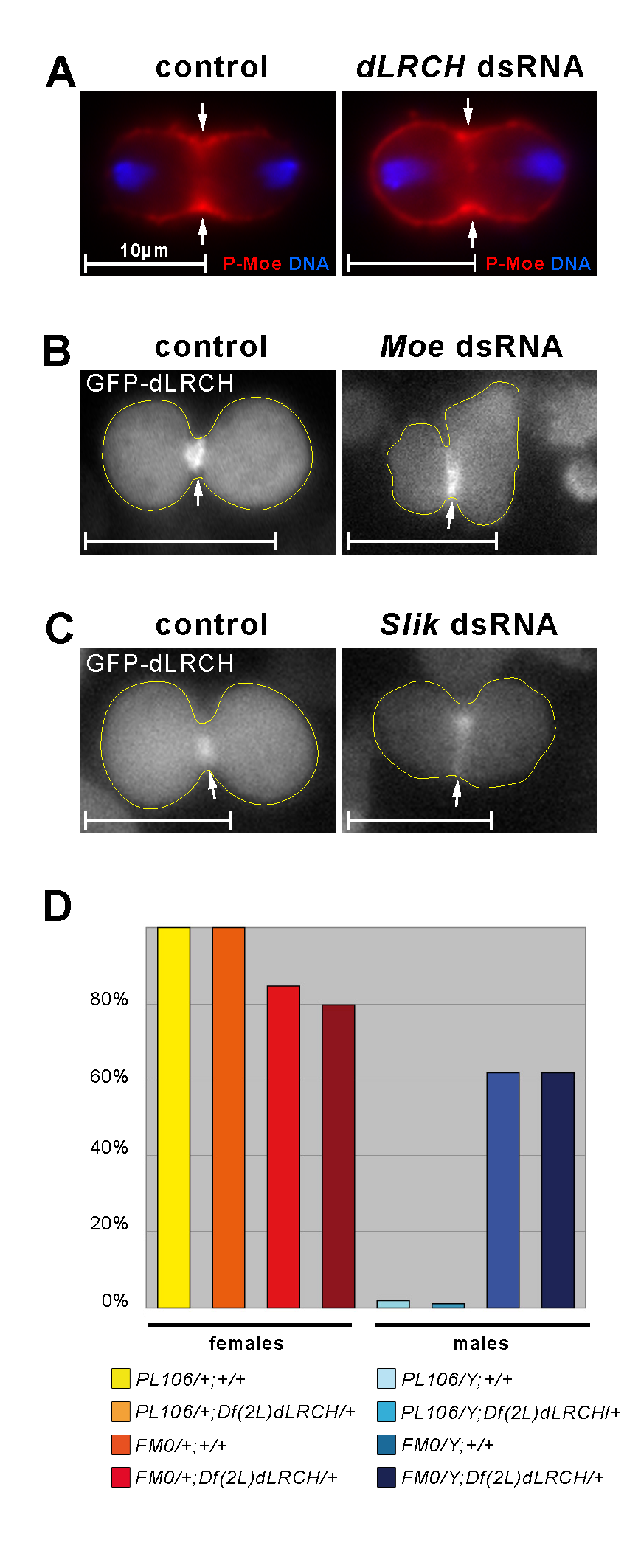

Supplement: Figure S3 — Reciprocal independence of dLRCH and the Moe pathway. A. Depletion of dLRCH does not affect P-Moe (red) distribution in dividing S2 cells, as shown by a normal accumulation of P-Moe at the equatorial cortex (arrows) during anaphase (in 91.4% of control cells, n = 58, and in 90% of dLRCH-depleted cells, n = 60). DNA is in blue B. Time-lapse frames of GFP-dLRCH S2 cells in control (left), and after treatment with Moe dsRNA (right) show that, reciprocally, Moe depletion does not prevent the proper distribution of dLRCH, as shown by accumulation at the cleavage furrow (arrow) in 98.0% of control cells and 97.7% of Moe-depleted cells, n = 300. C. Time-lapse frames of GFP-dLRCH S2 cells show that Slik depletion does not prevent dLRCH localization at the cleavage furrow (arrow), with GFP-dLRCH accumulating at furrow in 93.3% of control cells and 92.0% of Slik-depleted cells, n = 150. D. The graph shows the proportion of the different genotypes, observed in the progeny from MoePL106/FMO females crossed with control or Df(2L)dLRCH homozygous males. The absence of a dLRCH allele does not modify the proportion of the different classes, including that of MoePL106 male escapers, when compared to control. The number of individuals counted was MoePL106/+: n = 641; MoePL106/+; Df(2L)dLRCH/+: n = 680; FM0/+: n = 543; FM0/+; D(2L)dLRCH/+: n = 541; MoePL106/Y: n = 12; MoePL106/Y; Df(2L)dLRCH/+: n = 8; FM0/Y: n = 397; FM0/Y; D(2L)dLRCH/+: n = 421. (5.57 MB TIF) [file pone.0012257.s003.tif]

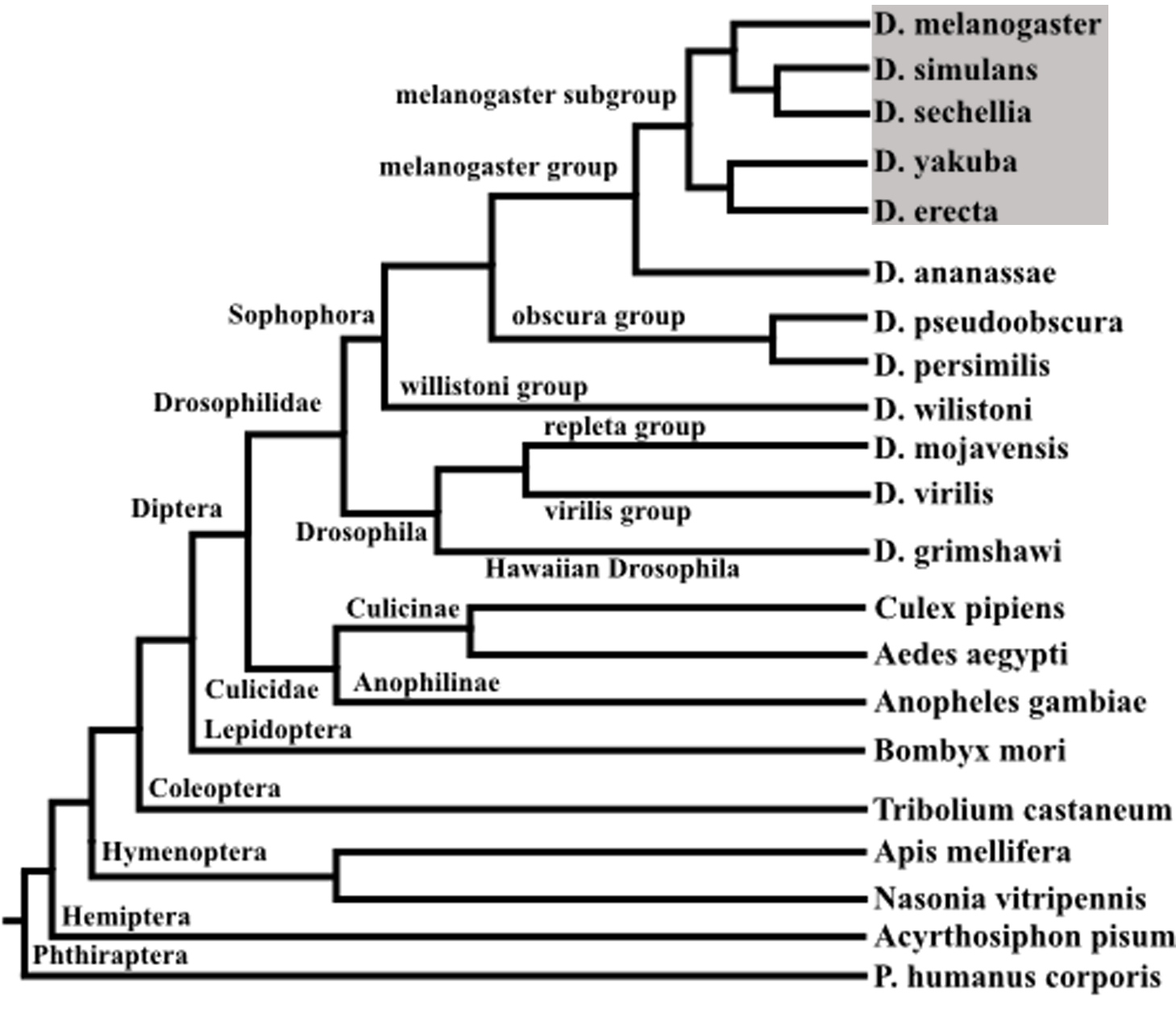

Supplement: Figure S4 — Evolution of the putative CG31804 gene in insect species. CG31804 putatively encodes a 212 amino-acid long peptide, being evolutionary conserved only in species of the melanogaster subgroup (grey box). Identities with the CG31804 encoded peptide decrease from 77% (D. simulans) to 57% (D. erecta). In the D. Ananassae genome, a genomic region at approximately the same location was identified by DNA similarity, but the presumptive ORF is interrupted by several stop codons and peptides do not display homology with the CG31804 product deduced from species of the melanogaster subgroup. Corresponding genome regions in other related drosophila were analysed with alignment tools (Blast, ClustalW2), which detect no significant DNA nor protein similarities. (6.46 MB TIF) [file pone.0012257.s004.tif]

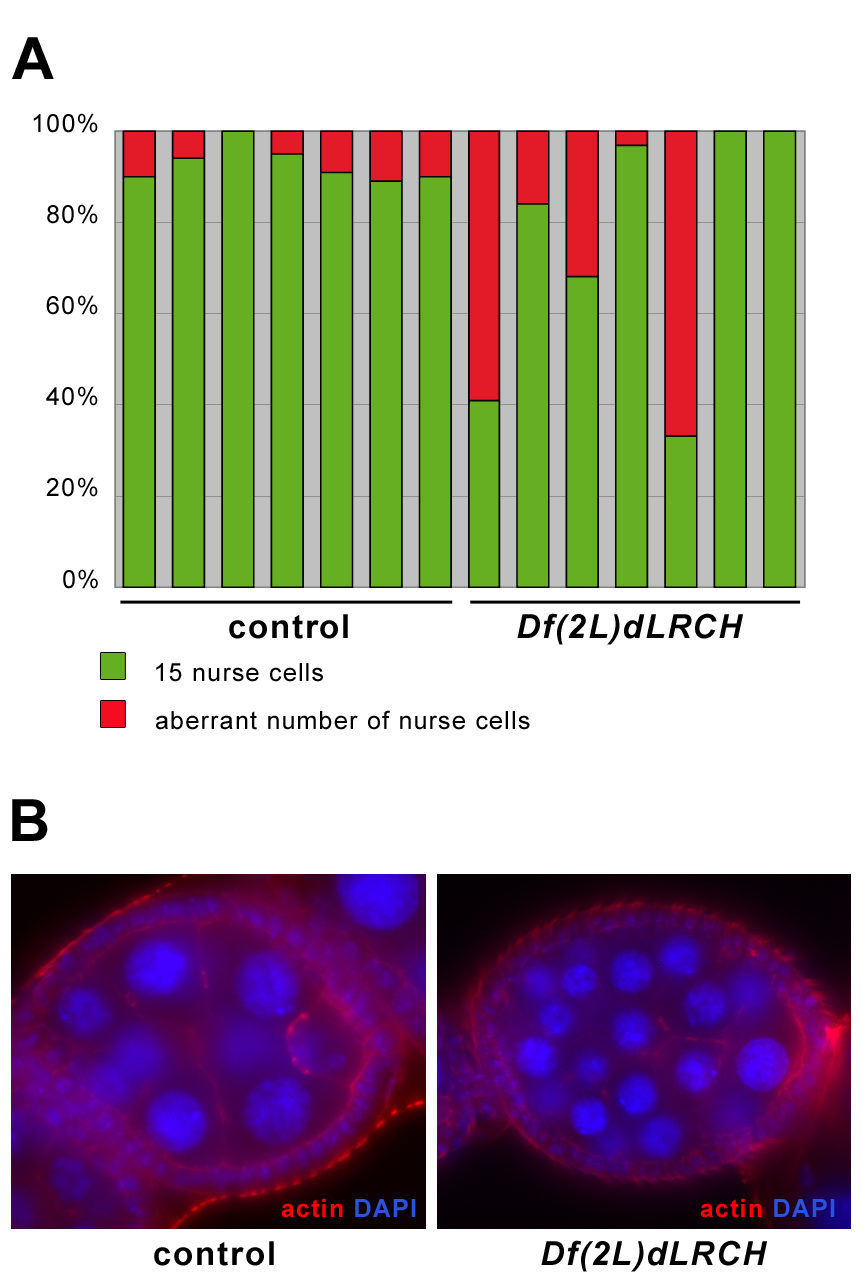

Supplement: Figure S5 — Germline defects observed in Df(2L)dLRCH females. A. Quantification of the number of nurse cells in the egg chambers of control or Df(2L)dLRCH homozygous individuals, in 7 independent experiments. Control (w1118) flies showed a weak proportion of abnormal numbers of nurse cells (<15, or >15, per egg chamber), in all experiments. In contrast we observed high variability in the proportion of abnormal egg chambers in Df(2L)dLRCH homozygous females. In two separate experiments, these defects were seen in 60% of examined samples. B. Pictures of egg chambers dissected from control (left) and Df(2L)dLRCH (right) females illustrating the observed defects in the number of nurse cells. Nuclei (blue) were stained by Dapi and F-actin (red) by Phalloidin-Texas-Red. (3.36 MB TIF) [file pone.0012257.s005.tif]

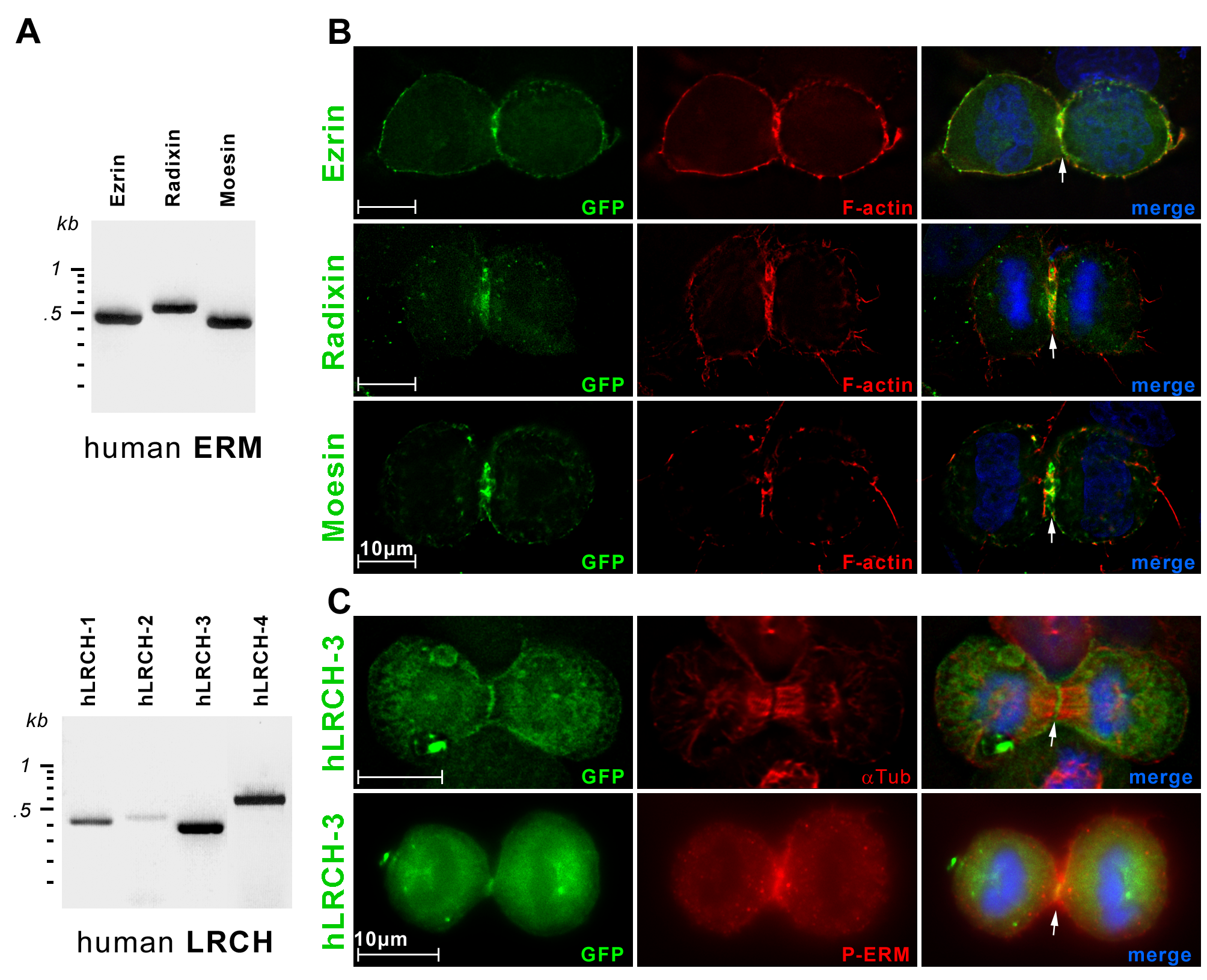

Supplement: Figure S6 — Expression and localization of human ERMs and LRCH3 in Hela cells. A. RT-PCR experiments for each of the 3 ERM and the 4 hLRCH mRNA show that all seven genes are expressed in HeLa cells. B. Sub-cellular localization of Ezrin-GFP, Radixin-GFP or Moe-GFP (green) and F-actin (red) during telophase indicates that all three human ERM proteins are located at the cleavage furrow (arrows). C. At the end of mitosis, GFP-hLRCH3 (green) is detected at the cleavage furrow as shown in co-labeling with α-Tubulin or P-ERM (red). DNA is in blue in merged images. (2.02 MB TIF) [file pone.0012257.s006.tif]
